# Let-7b regulates the expression of the growth hormone receptor gene in deletion-type dwarf chickens

**DOI:** 10.1186/1471-2164-13-306

**Published:** 2012-07-10

**Authors:** Shumao Lin, Hongmei Li, Heping Mu, Wen Luo, Ying Li, Xinzheng Jia, Sibing Wang, Xiaolu Jia, Qinghua Nie, Yugu Li, Xiquan Zhang

**Affiliations:** 1 Guangdong Provincial Key Lab of Agro-Animal Genomics and Molecular Breeding, Guangzhou, Guangdong, 510642, China; 2 Department of Animal Genetics, Breeding and Reproduction, College of Animal Science, South China Agricultural University, Guangzhou, Guangdong, 510642, China; 3 College of Veterinary Science, South China Agricultural University, Guangzhou, Guangdong, 510642, China; 4 College of Life Science, Foshan University, Foshan, Guangdong, 528231, China

## Abstract

**Background:**

A deletion mutation in the growth hormone receptor (*GHR*) gene results in the inhibition of skeletal muscle growth and fat deposition in dwarf chickens. We used microarray techniques to determine microRNA (miRNA) and mRNA expression profiles of *GHR* in the skeletal muscles of 14-day-old embryos as well as 7-week-old deletion-type dwarf and normal-type chickens. Our aim was to elucidate the miRNA regulation of *GHR* expression with respect to growth inhibition and fat deposition.

**Results:**

At the same developmental stages, different expression profiles in skeletal muscles of dwarf and normal chickens occurred for four miRNAs (miR-1623, miR-181b, let-7b, and miR-128). At different developmental stages, there was a significant difference in the expression profiles of a greater number of miRNAs. Eleven miRNAs were up-regulated and 18 down-regulated in the 7-week-old dwarf chickens when compared with profiles in 14-day-old embryos. In 7-week-old normal chickens, seven miRNAs were up-regulated and nine down-regulated compared with those in 14-day-old embryos. In skeletal muscles, 22 genes were up-regulated and 33 down-regulated in 14-day-old embryos compared with 7-week-old dwarf chickens. Sixty-five mRNAs were up-regulated and 108 down-regulated in 14-day-old embryos as compared with 7-week-old normal chickens. Thirty-four differentially expressed miRNAs were grouped into 18 categories based on overlapping seed and target sequences. Only let-7b was found to be complementary to its target in the 3′ untranslated region of *GHR*, and was able to inhibit its expression. Kyoto Encyclopedia of Genes and Genomes pathway analysis and quantitative polymerase chain reactions indicated there were three main signaling pathways regulating skeletal muscle growth and fat deposition of chickens. These were influenced by let-7b-regulated *GHR*. Suppression of the cytokine signaling 3 (*SOCS3*) gene was found to be involved in the signaling pathway of adipocytokines.

**Conclusions:**

There is a critical miRNA, let-7b, involved in the regulation of *GHR*. *SOCS3* plays a critical role in regulating skeletal muscle growth and fat deposition *via* let-7b-mediated *GHR* expression.

## Background

The complete growth and development of chickens is mainly dependent on the “hypothalamus-pituitary-target organ” pathway [1,2]. Depending on the needs of the body, the hypothalamus secretes growth hormone-releasing hormone and somatostatin. These play dual roles in the modulation and control of pituitary and growth hormone (GH) secretion [3,4]. GH circulates back to the liver *via* the blood and complexes with the GH receptor (GHR) on the liver cell surface to initiate intracellular signaling mechanisms that promote the expression of insulin-like growth factors (IGFs). IGFs circulate to the local tissues of the body through the bloodstream and promote cell growth and differentiation [5].

Skeletal muscle is the major target organ of GH. GH can act directly on the GHRs of skeletal muscle, producing paracrine and autocrine IGF-1 to regulate muscle growth and development [6,7]. Hodik and Vasilatos-Younken *et al.* showed that chicken GH can affect skeletal muscle cell proliferation and differentiation, regulates skeletal muscle abundance, and is involved in muscle metabolic regulation [8,9]. GHR is part of the GH-GHR-IGF growth axis, which regulates the expression of IGFs by mediating GH. Thus, it plays a role in regulating skeletal muscle growth and development.

Studies indicate that the sex-linked dwarf chicken (SLD) phenotype is caused by a mutation in the *GHR* gene. Point and deletion mutations, structural gene mutations, and mutations within the *GHR* regulatory region are all thought to be involved in conferring the SLD phenotype [10-13]. Of all these types of mutations, the deletion mutation is believed to be the main cause of this phenotype. Agarwal *et al.* found that that the deletion mutation exhibited a 1.7-kb deletion between exon 10 and the 3' untranslated region (3' UTR) of *GHR*[10]. The mutation results in a decrease in the number of muscle fibers and fiber diameter [11]. Dwarf chickens also present with increased carcass lipid content, which could be a result of increased lipogenesis and decreased energy expenditure [14]. Another study suggested that in laying hens, dwarfism reduces the adipose tissue lipid mobilization and likely also reduces *de novo* lipogenesis in the liver [15]. Expression of *GHR* mRNA is significantly up-regulated in dwarf chickens compared with normal chickens [16].

Dwarf phenotypes have been found in humans, mice, cattle, pigs, and other mammals [2,17-19]. Among them, the most studied is Laron syndrome in humans. Laron syndrome is familial dwarfism that was first reported in 1966, in which the serum GH level is normal, but IGF-1 levels are low [17]. Many studies have indicated that most cases of human Laron syndrome are caused by defects in *GHR*. Various types of mutations have been noted in *GHR*, leading to GHR extra-cellular domain inactivation. All of these can affect the binding of GHR and GH, and leads to interruption of GH signal transduction and a subsequent inability for GH to play its normal role [20-29].

Karen *et al.* found that the lifespan of mice was significantly prolonged after *GHR* was knocked out, but that growth was retarded. While IGF-1, IGFBP-1, IGFBP-3, and IGFBP-4 levels were significantly lower, IGFBP-2 levels were significantly increased, indicating that *GHR* defects led to GH signal transduction obstruction, significantly affecting phenotype [30]. Mavalli *et al.* found that defective skeletal muscle development in both *GHR* and *IGF-1R* mutants was attributable to diminished myoblast fusion and associated with compromised nuclear factor import and activity in activated T cells. Both mutants exhibited impaired skeletal muscle development, characterized by reductions in myofiber number and area as well as accompanying deficiencies in functional performance [31].

The above studies indicated that mutations in *GHR* could lead to the obstruction of normal human and animal skeletal muscle growth and fat deposition by causing GH signal transduction obstruction. However, the molecular mechanisms underlying the expression of *GHR* and its regulation of chicken skeletal muscle growth and fat deposition remain unclear.

Recently microRNAs (miRNAs) have been reported to be widespread endogenous noncoding RNA molecules involved in the regulation of gene expression [32,33]. In cells, miRNAs pair with a complementary target sequence in target mRNA 3' UTR to mediate the regulation of target gene expression [34]. These miRNAs are thought be involved in a series of important life processes, including development, neural differentiation, cell proliferation, cell apoptosis and fat metabolism [35]. Using the loss- and gain-of-function method, Kwon *et al.* showed that miRNA-1 of the ancient muscle-specific gene in *Drosophila* regulates functions of the heart and muscle-specific genes *via* their interaction with members of the Notch signaling pathway [36]. Clop *et al.* found that a point mutation within the 3' UTR of *GDF8* in Texel sheep resulted in a target site that allowed miR-1 and miR-206 to act simultaneously. This caused a reduction in the expression of the miRNA-mediated myostatin gene (*MSTN*) post-transcriptionally, leading to muscle hypertrophy [37]. Chen *et al.* demonstrated that miR-1 promotes differentiation of myoblasts into mature muscle cells by acting on *HDAC4*, inhibiting myoblast proliferation. The miRNA miR-133 promotes myoblast proliferation through the *SRF* gene, inhibiting myoblast differentiation [38]. These studies have suggested that there is further scope for understanding molecular mechanisms that regulate *GHR* expression.

In our study, we applied microarray technology to determine the miRNA and mRNA expression profiles in the skeletal muscles of dwarf and normal chickens at different stages of development. Critical miRNAs associated with *GHR* expression and the ways in which they regulate skeletal muscle growth and fat deposition were identified.

## Results

### Differential miRNA expression profiles in skeletal muscle of dwarf and normal chickens

Using signal values greater than 32 as the standard, a total of 124 miRNAs were detected in 22.9% of skeletal muscles of 14-day-old embryos from dwarf chickens. In normal chickens, 125 miRNAs were detected at a rate of 23.1%. At 7 weeks of age, 115 miRNAs were detected in the skeletal muscles of dwarf chickens at a detection rate of 21.2%, with 116 miRNAs detected in the skeletal muscles of normal chickens (21.4%).

Expression profiles were significantly different in four miRNAs between the two different types of chickens (Table 1). In 14-day-old embryos, expression of both miR-1623 and miR-181b was significantly down-regulated in dwarf chickens compared with normal chickens. Expression of let-7b and miR-128 in 7-week-old chickens was significantly up-regulated and down-regulated, respectively.

**Table 1:** The miRNA differential expression profiles of 14-day-old embryos and 7-week-old chickens skeletal muscle of dwarf and normal chickens

**Developmental stage**	**miRNA**	**Normal chickens**	**Dwarf chickens**	**Fold changes**	**p-value**
14-day-old embryos	miR-1623	4,755 ± 837	2,184 ± 302	2.18	8.53E-03
	miR-181b	7,741 ± 900	5,284 ± 588	1.46	2.76E-02
7-week-old chickens	let-7b	6,137 ± 345	6,992 ± 299	0.88	4.77E-02
	miR-128	1,455 ± 108	1,177 ± 99	1.24	4.87E-02

NoteNote: Fold change means the change in let-7b’s expression as compared normal with dwarf chickens.

### Differential miRNA expression profiles in skeletal muscle at different developmental stages

Expression of 11 miRNAs was up-regulated and 18 miRNAs down-regulated in 7-week-old dwarf chickens as compared with 14-day-old embryos. Expression of seven miRNAs was up-regulated and nine miRNAs down-regulated in normal 7-week-old chickens compared with 14-day-old embryos. Of the differentially expressed miRNAs, expression of let-7b, miR-30a-5p, miR-30b, miR-99a, and miR-133b was commonly up-regulated in both dwarf and normal chickens. The expression of miR-16c, miR-92, miR-106, miR-203, miR-451, and miR-454 was commonly down-regulated (Table 2).

**Table 2:** Differential miRNA expression profiles of skeletal muscle at different developmental stages as compared the 7-week-old chickens with the 14-day-old embryos

**miRNA**	**Dwarf chickens**	**Normal-type chickens**
Up-regulated	**let-7b**, miR-24, **miR-30a-5p**, **miR-30b**, miR-30d, miR-99a, miR-100, miR-133a, **miR-133b**, miR-133c, miR-146b	**let-7b**, **miR-30a-5p**, **miR-30b**, miR-30c, miR-99a, miR-126, **miR-133b**
Down-regulated	miR-15c, **miR-16c**, miR-17-5p, miR-20a, miR-20b, miR-21, **miR-92**, **miR-106**, miR-130b, miR-181b, miR-200b, **miR-203**, miR-205a, miR-206, **miR-451**, **miR-454**, miR-1576, miR-1777	miR-16, **miR-16c**, **miR-92**, **miR-106**, miR-199*, **miR-203**, **miR-451, miR-454**, miR-1579

### Differential mRNA expression profiles in skeletal muscle of dwarf and normal chickens

A total of 38,535 probes were used to detect mRNA, of which the probes displaying hybridization signals represented approximately 42.6–45.6% of the total. Probes lacking hybridization signals represented approximately 52.8–55.7% of the total, with 1.5–1.7% ambiguous hybridization signals. Using the normal chickens as a control group, screening of the differentially expressed genes in skeletal muscles was carried out using Significance Analysis of Microarrays (SAM) software. The screening criteria for signaling pathway analysis were that the q-value (%) was less than 5% and it showed a fold-change less than 2 (Additional file 1: Table S1).

The differential profiles in the skeletal muscle mRNA of the 14-day-old embryos showed that there were 55 genes with a greater than 2-fold change in differential expression between the dwarf and normal chickens. Of these, 33 were up-regulated and 22 were down-regulated. At 7 weeks, 173 genes had a greater than 2-fold change in differential expression between dwarf and normal chickens, with 108 mRNAs up-regulated and 65 down-regulated.

Further comparisons between 14-day-old embryos and 7-week-old normal and dwarf chickens indicated consistent up-regulation in the mRNA expression levels of five genes: *ARNT*, *BEAN*, *HSCB*, *LOC770114*, and *RCJMB04_1j22*. There were three genes, *GHR*, *LOC772190*, and *TMEM70* that presented with consistent down-regulation in normal chickens but consistent up-regulation in dwarf chickens. The mRNA expression of *GHR* in 14-day-old embryos of dwarf chickens was up-regulated 3.57-fold compared with normal chickens, and was up-regulated 5.26-fold in 7-week-old dwarf chickens as compared with normal chickens.

### Analysis of miRNA target genes and differentially expressed mRNA genes

Among the 34 differentially expressed miRNAs, there were some with the same seed sequence and target genes. This allowed for the classification of the miRNAs into 18 categories. There were another five miRNAs in which the target genes had not yet been discovered. The prediction results of the various types of differentially expressed miRNA target genes are shown in Additional file 2: Table S2.

We compared various differentially expressed miRNA prediction target genes with differentially expressed mRNA of genes from 7-week-old chicken skeletal muscle. In total, corresponding differentially expressed genes were found for 14 types of miRNAs (Additional file 3: Table S3). *GHR* was affected by let-7b, miR-15c, miR-16, and miR-16c.

### Let-7b-mediated regulation of *GHR* expression

The miRNAs involved in the regulation of *GHR* were let-7b, miR-15c (miR-16, miR-16c), and miR-181b (Additional file 3: Table S3). BLAST analysis indicated that the last 29 bp of the *GHR* 3' UTR exactly coincided with the target site of let-7b. This is consistent with the proposed mechanism of miRNAs as mainly targeting the 3' UTR of target genes. However, the target sites of miR-15c, miR-16, miR-16c and miR-181b were far apart from the *GHR* region. Expression levels of let-7b were significantly up-regulated in both dwarf and normal chickens at both stages of development investigated (Table 2).

### Signaling pathway analysis of let-7b-regulated *GHR*

Assuming that the dwarf chicken phenotype in this experiment was caused by a deletion mutation in *GHR*, we used the Kyoto Encyclopedia of Genes and Genomes (KEGG) software (http://www.genome.jp/kegg/) to conduct a pathway analysis for *GHR*. The results indicated that *GHR* is involved in the JAK-STAT signaling pathway (Additional file 4: Figure S1).

The 111 genes involved in the JAK-STAT signaling pathway have been summarized in Additional file 4: Figure S1. These genes were compared with the differentially expressed mRNAs in 7-week-old skeletal muscles from dwarf and normal chickens. It was found that only one gene, *SOCS3*, appeared in the mRNA expression profiles. The KEGG software was also used to analyze the signaling pathway of *SOCS3* and was found to be involved in the adipocytokine signaling pathway (Additional file 5: Figure S2).

From the adipocytokine signaling pathway, it can be seen that *SOCS3* influences cellular regulation in three ways: inhibiting the *IRS1* gene through inhibition of the phosphorylation of tyrosine in insulin receptor substrate 1 (IRS1) and thus inhibiting the insulin signalling pathway; inhibiting the *LEPR* gene; and inhibiting the *JAK* gene.

### Quantitative polymerase chain reaction (qPCR) analysis of the *GHR* signaling pathway regulation by let-7b

**Figure 1:**
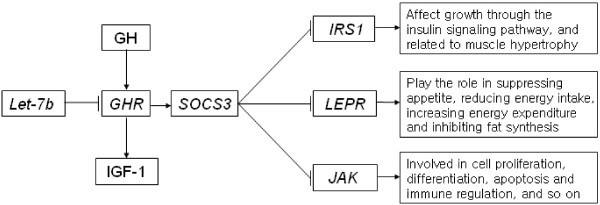
**Schematic illustration of the signaling pathway of*GHR*regulated by let-7b.** miRNA let-7b inhibits the expression of *GHR* and regulates *SOCS3***.***SOCS3* regulates the growth and development of chickens through three signaling pathways: inhibiting the phosphorylation of tyrosine in *IRS1*; inhibiting *LEPR*; and inhibiting *JAK*. *IRS1*, *LEPR* and *JAK* are involved in growth, fat synthesis, and cell proliferation.

**Figure 2:**
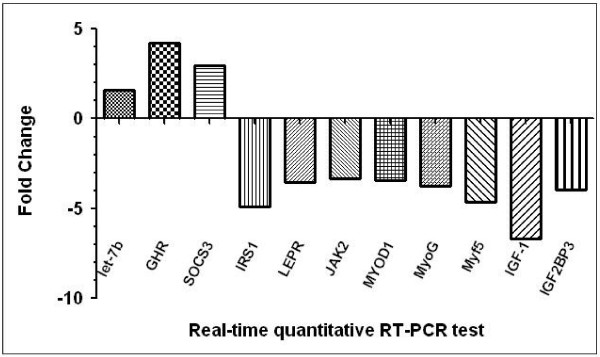
**Validation of differentially expressed mRNA.** When comparing dwarf and normal chickens, the expression levels of let-7b were significantly up-regulated. The mRNA expression levels of *GHR* and *SOCS3* were significantly up-regulated, but those of *IRS1, LEPR* and *JAK2* are significantly down-regulated. All mRNA expression levels of *MYOD1*, *MyoG* and *Myf5* regulating the growth of skeletal muscle were significantly up-regulated in normal chickens, and significantly down-regulated in dwarf chickens. The mRNA expression levels of *IGF1* and *IGF2BP3*, associated with the insulin signaling pathway, were significantly down-regulated in dwarf chickens.

Based on the previously mentioned analyses, a schematic illustration of the *GHR* signaling pathway, as regulated by let-7b, was developed (Figure 1). To verify the signaling pathway, we carried out qPCR assays to determine mRNA expression levels in the skeletal muscles of 7-week-old dwarf and normal chickens. The qPCR analysis showed that mRNA expression of *GHR* and *SOCS3* was up-regulated 4.24- and 2.95-fold, respectively in dwarf chickens compared with normal chickens. The mRNA expression of these genes was up-regulated 5.16- and 2.67-fold respectively, as shown by microarray analysis. Expression of *IRS1*, *LEPR* and *JAK2* was down-regulated 4.92-, 3.53- and 3.32-fold, respectively. The expression of *MYOD1*, *MyoG* and *Myf5*, which regulate skeletal muscle growth, was down-regulated 3.46-, 3.78- and 4.66-fold, respectively, in dwarf chickens compared with normal chickens. In addition, expression of *IGF1* and *IGF2BP3* in the insulin pathway is also down-regulated 6.73- and 3.97-fold respectively, in dwarf compared with normal chickens (Figure 2).

### Validation of the 3′ UTR of *GHR* as the target site of let-7b

**Figure 3:**
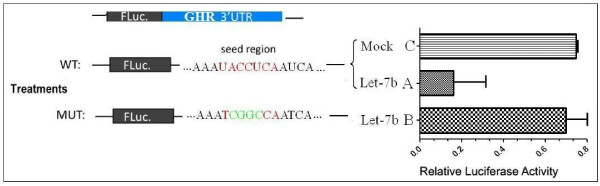
**Dual-luciferase reporter assay for validation of the 3′ UTR of*GHR*as the target site of the let-7b*in vitro*cell system.** DF-1 cells were transfected with pmirGLO-let-7b-*GHR* 3′ UTR, pmirGLO-let-7b-*GHR* 3′ UTR mutation plasmid, or the control plasmid. The activity of firefly luciferase (luc2) and Renilla luciferase (hRluc-neo) was measured and relative values (luc2/hRluc-neo) calculated.

Luciferase activity was decreased in DF-1 cell lines transfected with pmirGLO-let-7b-*GHR* 3′ UTR (Figure 3A), but increased in DF-1 cells transfected with pmirGLO-let-7b-*GHR* 3′ UTR mutation, and in the control DF-1 cell line (Figure 3 B and C). This confirms that the expression of *GHR* is regulated by let-7b, and that the let-7b target is located in the region of the *GHR* 3′ UTR mutation.

**Figure 4:**
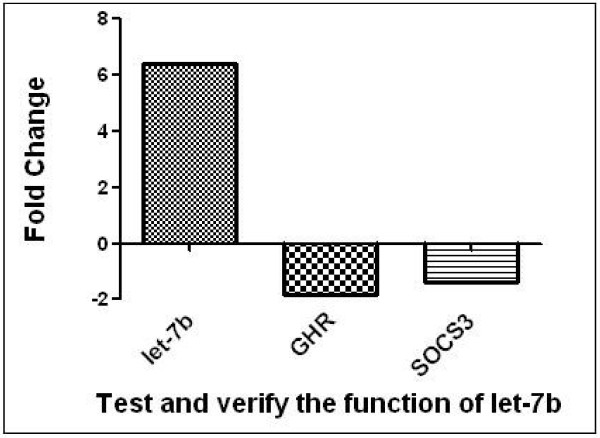
**Functional validation of let-7b inhibiting*GHR*in an*in vitro*cell system.** pcDNA-EGFP-pre-let-7b was transfected into the DF-1 cell line compared with cells transfected with the control plasmid.

In the overexpression assay for let-7b, we observed it was up-regulated 6.41-fold as compared with the control. Conversely, expression of *GHR* was down-regulated 1.82-fold. Additionally, expression of *SOCS3*, stimulated by *GHR*, was down-regulated 1.39-fold (Figure 4).

## Discussion

miRNAs are a class of non-coding small RNA molecules with a length of 18–24 nucleotides. They can direct the regulation of the expression levels of certain genes, control cell growth and development, and determine tissue type during cell differentiation by reducing the stability of target genes or inhibiting translation levels to influence cell differentiation, proliferation, and apoptosis. In animal cells, miRNAs, by interacting with a specific sequence of target gene mRNA, inhibit protein synthesis or induce mRNA degradation and post-transcriptionally negatively regulate the expression of target genes [39,40].

In this study, high-throughput microarray technology was used to analyze miRNA and mRNA expression profiles in the skeletal muscles of 14-day-old embryos and 7-week-old dwarf and normal chickens to identify miRNAs related to skeletal muscle growth and development. In chickens, 499 pre-miRNAs and 544 mature-miRNAs have been reported [41,42]. In the present study, 124 and 125 miRNAs were detected in the skeletal muscles of 14-day-old embryos from dwarf and normal chickens, respectively. We also detected 115 and 116 miRNAs in the skeletal muscles of 7-week-old dwarf and normal chickens, respectively. Such tissue-specific miRNA expression has been reported in a few previous studies [41,43-46]. Our data also showed that there is significantly different expression for only a few miRNAs at the same developmental stages in dwarf and normal chickens. However, the expression profiles of a greater number of miRNAs at different developmental stages for dwarf and normal chickens were significantly different. When comparing 7-week-old chickens with 14-day-old embryos, more down-regulated miRNAs than up-regulated miRNAs were detected. This would suggest that down-regulated expression of miRNAs is favorable for muscle growth and development in chickens at 7 weeks. In 7-week-old chickens, as compared with 14-day-old embryos, the expression of let-7b, miR-30a-5p, miR-30b, miR-99a and miR-133b was significantly up-regulated, but miR-16c, miR-92, miR-106, miR-203, miR-451 and miR-454 were significantly down-regulated in both dwarf and normal chickens. Considering that *GH* and *GHR* play important roles in chicken growth and development, we focused on observing the miRNAs involved in the regulation of their expression.

Four miRNAs, let-7b, miR-16, miR-16c, and miR-181b, are involved in the regulation of *GHR*. BLAST analysis confirmed that the target location of let-7b was in the deleted region of *GHR* 3' UTR. But the target locations of miR-16, miR-16c and miR-181b were distant from the deleted region. We concluded that the regulation of let-7b could be critical to *GHR* expression. As the deletion mutation in dwarf chickens results in the loss of the ability of let-7b to pair with sequences in its target gene, the regulation of growth and development is affected.

Skeletal muscle growth and development in chickens is fastest at the 7-week-old stage; conversely, the growth and development of skeletal muscle during the embryonic period is relatively slow. Comparing dwarf with normal chickens, there was significantly different mRNA expression for 173 genes in the 7-week-old chickens; however, there was significantly different mRNA expression for only 55 genes in the 14-day-old embryos. For both 14-day-old embryos and 7-week-old chickens, mRNA expression of *GHR* was up-regulated 3.57- and 5.26-fold, respectively, in dwarf chickens compared with normal chickens. It is suggested that the mRNA corresponding to *GHR* was inhibited in normal chickens as reported by Wu *et al.*[16].

Comparing the different developmental stages (Table 2), expression levels of let-7b were significantly up-regulated in both dwarf and normal chickens. *GHR* expression was up-regulated in dwarf chickens and down-regulated in normal chickens, suggesting that let-7b could play a significant role in inhibiting *GHR* expression, further promoting the growth and development of skeletal muscle.

The let-7b miRNA is a member of the let-7 family. Deletion, or mutation of the function of let-7, may lead to defects in the transformation of nematodes from their larval to adult stage [44]. Methylation, post-translation modifications, and Lin28 genes regulate the let-7 family. Additionally, the family regulates *RASMYCHMAG2CDC25ACDK2*, and other target genes that influence a variety of biological phenomena and physiological processes, especially during biological development, cell proliferation and differentiation, and tumor suppression. There are 13 homologs in the let-7 family in the human genome, clustered into eight sites [45]. These gene clusters are located at fragile sites related to lung cancer, breast cancer, urothelial cancer, and cervical cancer, suggesting that they may act as tumor suppressors. Previous studies of the let-7 family have largely focused on tumor suppression mechanisms [46], and studies investigating the family’s role in growth and development are rare.

The signaling pathway related to the regulation of the growth and development of skeletal muscle by let-7b-mediated *GHR* has not been previously reported. GH plays important roles in regulating animal growth and development, and its action on tissues and cells is mediated through its binding with GHR on the cell surface. GHR is activated upon binding of GH to stimulate the growth and metabolism of muscle, bone, and cartilage cells [3,4]. GH also regulates chicken growth through close binding to its receptor and activating expression of IGF. The amount and action of GHR has direct effect on GH physiological function. In the present study, the mRNA expression of *GHR* was significantly up-regulated in dwarf chickens compared with normal chickens. The up-regulated mRNA expression of *GHR* retarded chicken growth, probably owing to a certain compensation mechanism [16]. Our data showed that the retarded growth of dwarf chickens was caused by a deletion in the *GHR* 3′ UTR inducing loss of the let-7b target site. Through signaling pathway analysis, we found that let-7b regulates the expression of *GHR*, and further regulates *SOCS3* through the JAK-STAT signaling pathway. Studies have shown that *SOCS3* can inhibit excessive cell growth and induce apoptosis as part of maintaining cell stability [47,48]. *SOCS3* regulates the growth and development of chickens through three adipocytokine signaling pathways. (1) *SOCS3* inhibits the tyrosine in *IRS1*. By inhibiting the phosphorylation of IRS1, *SOCS3* inhibits insulin signaling, thus affecting growth. (2) *SOCS3* inhibits *LEPR*, and up-regulated *SOCS3* expression in dwarf chickens may affect the function of leptin. Leptin has a wide range of biological effects, with an important role in the metabolic regulation center of the hypothalamus, which plays a role in suppressing appetite, reducing energy intake, increasing energy expenditure and inhibiting fat synthesis. This helps explain why dwarf chickens are more likely to be obese [14]. (3) *SOCS3* inhibits *JAK*; the JAK-STAT signaling pathway is a recently discovered signal transduction pathway stimulated by cytokines, and is involved in cell proliferation, differentiation, apoptosis, immune regulation, and many other important biological processes.

In the present study, little change in expression of let-7b between dwarf and normal chickens was observed; however, growth was retarded in dwarf chickens. In dwarf chickens, let-7b could not inhibit the expression of *GHR*. This allows for the gene to be up-regulated as let-7b is unable to pair with *GHR* gene as its target site is deleted. Data from the microarray and qPCR analyses supported that the above pathway, indicating that the expression of *GHR* is inhibited by let-7b, and the expression of *SOCS3* gene is regulated and stimulated by *GHR*. Further qPCR data supported that *SOCS3* could inhibit the expression of *IRS1*, *LEPR* and *JAK*. The expression of *IRS1*, *LEPR* and *JAK* was significantly down-regulated, expression of genes regulating skeletal muscle growth (*MYOD1*, *MyoG* and *Myf5*) and the insulin pathway (*IGF1* and *IGF2BP3*) were also down-regulated significantly.

## Conclusions

A comparison of dwarf chickens with normal chickens at the same developmental stages revealed that expression profiles of only a few miRNAs were significantly different. In 14-day-old embryos, the expression profiles of a greater number of miRNAs were significantly different compared with those in 7-week-old chickens. By combining target gene prediction for differential miRNAs, joint analysis of mRNA expression profiles, and BLAST analysis, the critical role of let-7b in regulating the *GHR* expression was identified. With the aid of KEGG signaling pathway and qPCR analyses, the network through which let-7b-mediated *GHR* regulates growth and development of skeletal muscle as well as fat deposition was established. It was confirmed that *SOCS3* plays a critical role in inhibiting *IRS1*, *LEPR*, and *JAK*.

## Methods

### Animals

Dwarf and normal recessive White Rock chickens, both bred for nearly 10 generations, were used. Dwarf chickens had a 1 773-bp deletion mutation at the end of exon 10 and in the 3' UTR of *GHR*. The two strains were fed under the same conditions to 7 weeks of age. The weight of dwarf chickens was about 30% less than that of normal chickens. Randomly selected embryos of dwarf chickens and normal chickens were incubated for 14 d, dissected, and their sex identified according to gonad development. Nine female embryos for each chicken strain were selected for leg muscle separation. Skin and bones were removed and the muscle divided into three parts. The divided parts were placed into cryopreservation tubes then quickly placed into liquid nitrogen (−196°C) for preservation. Nine dwarf and nine normal chickens, fed by conventional breeding methods until 7 weeks of age, were randomly selected, and their leg muscles separated. The central muscle of the gastrocnemius was taken and divided into three parts, placed into cryopreservation tubes and quickly placed into liquid nitrogen (−196°C) for preservation. All animal experiments involved in this study were approved by the Animal Care Committee of South China Agricultural University (Guangzhou, People's Republic of China). Chickens were euthanized as necessary to ameliorate suffering.

### Extraction of total RNA

Total RNA was isolated from 0.2 g of skeletal muscle tissues with TRIzol® (Invitrogen Life Technologies, Carlsbad, CA, USA) according to the manufacturer’s instructions using an RNeasy MinElute Cleanup Kit. All mRNA was quantified by spectrophotometry (ND-2000, NanoDrop Inc., USA). The purity and yield of RNA was determined using optical density at 260 and 280 nm. RNA integrity was examined by electrophoresis on a 1.2% denaturing formaldehyde gel.

### Microarray analysis

Three pools of RNA were prepared for each chicken strain, with each pool containing RNA from three individuals. The miRNA chips were designed based on miRNAs listed in miRBase Version 15.0 (http://www.sanger.ac.uk/Software/Rfam/mirna/), and prepared by LC Sciences (Houston, Texas, USA). The miRNA chips used in the present study contained a total of 542 miRNA sequences. Normalization of chip data was carried out using the Lowess (Locally-weighted Regression) method [49], and t-tests of the data were conducted following normalization. Microarray assays for miRNAs were performed using a service provider (LC Sciences, Houston, Texas, USA). Raw data were provided as ceiling exposure limits or Excel files for subsequent statistical analysis.

Hybridization was performed with 100 μL 6× SSPE buffer (0.90 M NaCl, 60 mM Na_2_HPO_4_, 6 mM EDTA, pH 6.8) containing 25% formamide at 34°C. Hybridization detection was facilitated using fluorescent tag-specific Cy3 and Cy5 dyes. Hybridization images were collected using a laser scanner (GenePix 4000B, Molecular Device) and digitized using Array-Pro image analysis software (Media Cybernetics). Twelve microarray data were MIAME compliant, and the raw data were deposited in a database (ArrayExpress, GEO) with the accession number GSE37360, GSE37367 and GSE37368. Data were analyzed by first subtracting the background, and then the signals were normalized using a LOWESS filter.

The raw microarray data set was filtered according to a standard procedure to exclude spots with minimum intensity. It was arbitrarily set to an intensity parameter of P300 for mRNA expression data, and P100 for the miRNA microarray data, on both fluorescence channels. If the fluorescence intensity of one channel was below the cut-off while the other was above, the lower channel intensity was overridden. Spots with diameters less than 25 μm for the cDNA expression array and less than 10 μm for the miRNA microarray and flagged spots were also excluded from the analyses. For two color experiments, the ratio of the two sets of detected signals (log2 transformed, balanced) and p-values of the *t*-test were calculated. Differentially detected signals were those with p-values less than 0.01. Any false correction tests were performed for microarray data by qPCR.

The detection of mRNA expression profiles using Affymetrix’s Chicken Genechip was completed by the Beijing Capital Bio Corporation (Beijing, China). The mRNA chip used in the present study contained a total of 38,535 probes. Each sample had three biological replicates, and SAM software was used for the analysis of differentially expressed genes. The screening criteria were as follows: q-value ≤ 5%; with a fold change ≥ 2; or a fold change ≤ 0.5.

### Correlation analysis between miRNA and mRNA expression profiles

We combined our miRNA expression data and mRNA expression data to generate a miRNA-mRNA interaction database using target gene mapping methods and MAS software. The miRNA and mRNA expression chip profile-associated analyses combined with network predictions, estimates the target genes of differentially expressed miRNAs. To further validate microarray results, we performed qPCR experiments for representative genes. The target genes of these miRNAs were identified by qPCR.

### Target gene prediction

TargetScan 5.1 (http://www.targetscan.org/) was used to carry out target gene prediction for the differentially expressed miRNA. TargetScan 5.1 proposed the concept of the ‘seed region’, increasing prediction accuracy, was the software with the lowest false positive rate for predicting miRNA targets.

### miRNA target gene and mRNA differential expression profiles

Differentially expressed miRNA prediction target gene sets were compared with the mRNA differential expression profiles from 7-week-old chicken skeletal muscle, and the target genes affected by miRNA were selected.

### Signaling pathway analysis

KEGG is a bioinformatics database established by the Kanehisa Laboratory of the Japan Kyoto University Bioinformatics Centre [50,51]. KEGG links genome information with gene function, thereby linking genomic and functional information. In this study, the KEGG PATHWAY database (http://www.genome.jp/kegg/) software platform was used for signaling pathway analysis of *GHR* regulatory networks.

### qPCR analysis

Quantitative PCR was used to detect mRNA expression levels of the major genes in the signaling pathway. Using published genome sequences, the Primer Premier 5 software was used for primer design (Additional file 6: Table S4). In the present study, the Ct value was applied to detect the mRNA expression of the samples, and three replicates were set for each sample. The thermal cycling protocol was: 95°C for 1 min, then 40 cycles of 95°C for 15 s, appropriate annealing temperature for 45 s, and 72°C for 45 s. The final step after cycling was an extension at 72°C for 40 s. Melting curve analysis was carried out to determine the specificity of PCR products. The ΔΔCt method was used to measure gene expression with β-actin as the reference gene.

### Luciferase reporter assays

Based on the data in the miRBase bank (http://www.mirbase.org/), primers for amplifying pre-let-7b were designed (Additional file 7: Table S5). PCR products including pre-let-7b were ligated and transformed using the pEASY-T1 Simple Clone Kit (Trans Gen Biotech, Beijing, China). Two plasmids, pcDNA3.1-EGFP (Invitrogen) and pEASY-T1-pre-let-7b, were used for constructing the let-7b expression plasmid pcDNA3.1-EGFP-pre-let-7b. Based on the data in GenBank, primers for amplifying the *GHR* 3′ UTR region were designed (Additional file 7: Table S5). The plasmid pmirGLO-let-7b-*GHR* 3′ UTR was prepared for verification of *GHR* mRNA expression. Two types of plasmids, the wild-type, and a mutant with *GHR* deleted were prepared. Plasmids pcDNA-EGFP-pre-let-7b and pmirGLO-let-7b-*GHR* 3′ UTR were co-transfected into DF-1 cells (3 × 10^4^ cells). Validation of *GHR* as the target of let-7b, and luciferase reporter assays for functional validation *in vitro* were conducted. Expression levels of *GHR* and other correlated genes were measured using qPCR analysis *in vitro.*

## Competing interests

The authors declare that they have no competing interests.

## Authors’ contributions

S.L., H.M., W.L., Y.L., S.W. and X.J. carried out experiments. S.L., H.L. and X.J. analysed data. S.L., W.L. and X. Z. wrote the paper. S.L., H.L., Q.N., Y.L. and X.Z. designed experiments. All authors have read and approved the final manuscript.

## Supplementary Material

Additional file 1 **Table S1.** The skeletal muscle mRNA differential profile of 14-day-old embryos and 7-week-old chickens of normal chickens and dwarf chickens. Click here for file

Additional file 2 **Table S2.** The summary table of prediction results of the differentially expressed. Click here for file

Additional file 3 **Table S3.** The intersection genes of the differentially expressed genes of miRNA target genes and mRNA genes. Click here for file

Additional file 4 **Figure S1.** The JAK-STAT signaling pathway with GHR gene involved in KEGG links the genome information with gene function. The pathway includes 111 genes in total. Click here for file

Additional file 5 **Figure S2.** Adipocytokine signaling pathway with the SOCS3 gene involved in. Click here for file

Additional file 6 **Table S4.** Sequences of primers used for qRT-PCR. Click here for file

Additional file 7 **Table S5.** Sequences of primers used for vectors construction. Click here for file

## References

[B1] Schwartzbauer G Menon RK Regulation of growth hormone receptor gene expression Mol Genet Metab 1998 63 4 243 253 10.1006/mgme.1998.2685 9635292

[B2] Hull KL Harvey S Growth hormone resistance: clinical states and animal models J Endocrinol 1999 163 2 165 172 10.1677/joe.0.1630165 10556764

[B3] Porter TE Regulation of pituitary somatotroph differentiation by hormones of peripheral endocrine glands Domest Anim Endocrinol 2005 29 1 52 62 10.1016/j.domaniend.2005.04.004 15885962

[B4] Kühn ER Geelissen SM Van der Geyten S Darras VM The release of growth hormone (GH): relation to the thyrotropic- and corticotropic axis in the chicken Domest Anim Endocrinol 2005 29 1 43 51 10.1016/j.domaniend.2005.02.022 15927766

[B5] Pierce AL Fukada H Dickhoff WW Metabolic hormones modulate the effect of growth hormone (GH) on insulin-like growth factor-I (IGF-I) mRNA level in primary culture of salmon hepatocytes J Endocrinol 2005 184 2 341 349 10.1677/joe.1.05892 15684342

[B6] Etherton TD Bauman DE Biology of somatotropin in growth and lactation of domestic animals Physiol Rev 1998 78 3 745 761 9674693 10.1152/physrev.1998.78.3.745

[B7] Argetsinger LS Carter-Su C Mechanism of signaling by growth hormone receptor Physiol Rev 1996 76 4 1089 1107 8874495 10.1152/physrev.1996.76.4.1089

[B8] Hodik V Mett A Halevy O Mutual effects of growth hormone and growth factors on avian skeletal muscle satellite cells Gen Comp Endocrinol 1997 108 1 161 170 10.1006/gcen.1997.6964 9378270

[B9] Vasilatos-Younken R Wang XH Zhou Y Day JR McMurtry JP Rosebrough RW Decuypere E Buys N Darras V Beard JL Tomas F New insights into the mechanism and actions of growth hormone (GH) in poultry Domest Anim Endocrinol 1999 17 2–3 181 190 10527121 10.1016/s0739-7240(99)00035-1

[B10] Agarwal SK Cogburn LA Burnside J Dysfunctional growth hormone receptor in a strain of sex-linked dwarf chicken: evidence for a mutation in the intracellular domain J Endocrinol 1994 142 3 427 434 10.1677/joe.0.1420427 7964293

[B11] Knízetová H Effects of the sex-linked dwarf gene (dw) on skeletal muscle cellularity in broiler chickens Br Poult Sci 1993 34 3 479 485 10.1080/00071669308417603 8358635

[B12] Huang N Cogburn LA Agarwal SK Marks HL Burnside J Overexpression of a truncated growth hormone receptor in the sex-linked dwarf chicken: evidence for a splice mutation Mol Endocrinol 1993 7 11 1391 1398 10.1210/me.7.11.1391 8114754

[B13] Tanaka M Hayashida Y Wakita M Hoshino S Nakashima K Expression of aberrantly spliced growth hormone receptor mRNA in the sex-linked dwarf chicken, Gifu 20 Growth Regul 1995 5 4 218 223 8745148

[B14] Touchburn SP Guillaume J Leclercq B Blum JC Lipid and energy metabolism in chicks affected by dwarfism (dw) and Naked-neck (Na) Poult Sci 1980 59 10 2189 2197 10.3382/ps.0592189 7465494

[B15] Burghelle-Mayeur C Tixier-Boichard M Merat P Demarne Y De novo lipogenesis and lipolysis activities in normal (Dw) and dwarf (dw) White Leghorn laying hens Comp Biochem Physiol B 1989 93 4 773 779 10.1016/0305-0491(89)90044-8 2680251

[B16] Wu GQ Zheng JX Yang N Expression profiling of GH, GHR, and IGF-1 genes in sex-linked dwarf chickens Yi Chuan 2007 29 8 989 994 17681929 10.1360/yc-007-0989

[B17] Laron Z Pertzelan A Mannheimer S Genetic pituitary dwarfism with high serum concentation of growth hormone–a new inborn error of metabolism? Isr J Med Sci 1966 2 2 152 155 5916640

[B18] Hale CS Herring WO Shibuya H Lucy MC Lubahn DB Keisler DH Johnson GS Decreased growth in angus steers with a short TG-microsatellite allele in the P1 promoter of the growth hormone receptor gene J Anim Sci 2000 78 8 2099 2104 10947094 10.2527/2000.7882099x

[B19] Aggrey SE Yao J Sabour MP Lin CY Zadworny D Hayes JF Kuhnlein U Markers within the regulatory region of the growth hormone receptor gene and their association with milk-related traits in Holsteins J Hered 1999 90 1 148 151 10.1093/jhered/90.1.148 9987923

[B20] Amselem S Duquesnoy P Attree O Novelli G Bousnina S Postel-Vinay MC Goossens M Laron dwarfism and mutations of the growth hormone-receptor gene N Engl J Med 1989 321 15 989 995 10.1056/NEJM198910123211501 2779634

[B21] Edery M Rozakis-Adcock M Goujon L Finidori J Lévi-Meyrueis C Paly J Djiane J Postel-Vinay MC Kelly PA Lack of hormone binding in COS-7 cells expressing a mutated growth hormone receptor found in Laron dwarfism J Clin Invest 1993 91 3 838 844 10.1172/JCI116304 8450064 PMC288035

[B22] Berg MA Argente J Chernausek S Gracia R Guevara-Aguirre J Hopp M Pérez-Jurado L Rosenbloom A Toledo SP Francke U Diverse growth hormone receptor gene mutations in Laron syndrome Am J Hum Genet 1993 52 5 998 1005 8488849 PMC1682057

[B23] Freeth JS Ayling RM Whatmore AJ Towner P Price DA Norman MR Clayton PE Human skin fibroblasts as a model of growth hormone (GH) action in GH receptor-positive Laron's syndrome Endocrinology 1997 138 1 55 61 10.1210/en.138.1.55 8977385

[B24] Diniz ET Jorge AA Arnhold IJ Rosenbloom AL Bandeira F Novel nonsense mutation (p.Y113X) in the human growth hormone receptor gene in a Brazilian patient with Laron syndrome Arq Bras Endocrinol Metabol 2008 52 8 1264 1271 19169479 10.1590/s0004-27302008000800010

[B25] Fassone L Corneli G Bellone S Camacho-Hübner C Aimaretti G Cappa M Ubertini G Bona G Growth hormone receptor gene mutations in two Italian patients with Laron Syndrome J Endocrinol Invest 2007 30 5 417 420 17598975 10.1007/BF03346320

[B26] Ying YQ Wei H Cao LZ Lu JJ Luo XP Clinical features and growth hormone receptor gene mutations of patients with Laron syndrome from a Chinese family Zhongguo Dang Dai Er Ke Za Zhi 2007 9 4 335 338 17706034

[B27] Gennero I Edouard T Rashad M Bieth E Conte-Aurio F Marin F Tauber M Salles JP El Kholy M Identification of a novel mutation in the human growth hormone receptor gene (GHR) in a patient with Laron syndrome J Pediatr Endocrinol Metab 2007 20 7 825 831 17849745 10.1515/jpem.2007.20.7.825

[B28] Arman A Ozon A Isguven PS Coker A Peker I Yordam N Novel splice site mutation in the growth hormone receptor gene in Turkish patients with Laron-type dwarfism J Pediatr Endocrinol Metab 2008 21 1 47 58 18404972 10.1515/jpem.2008.21.1.47

[B29] Arman A Yüksel B Coker A Sarioz O Temiz F Topaloglu AK Novel growth hormone receptor gene mutation in a patient with Laron syndrome J Pediatr Endocrinol Metab 2010 23 4 407 414 20583548 10.1515/jpem.2010.064

[B30] Coschigano KT Holland AN Riders ME List EO Flyvbjerg A Kopchick JJ Deletion, but not antagonism, of the mouse growth hormone receptor results in severely decreased body weights, insulin, and insulin-like growth factor I levels and increased life span Endocrinology 2003 144 9 3799 3810 10.1210/en.2003-0374 12933651

[B31] Mavalli MD DiGirolamo DJ Fan Y Riddle RC Campbell KS van Groen T Frank SJ Sperling MA Esser KA Bamman MM Clemens TL Distinct growth hormone receptor signaling modes regulate skeletal muscle development and insulin sensitivity in mice J Clin Invest 2010 120 11 4007 4020 10.1172/JCI42447 20921627 PMC2964973

[B32] Bartel DP MicroRNAs: genomics, biogenesis, mechanism, and function Cell 2004 116 2 281 297 10.1016/S0092-8674(04)00045-5 14744438

[B33] Filipowicz W Bhattacharyya SN Sonenberg N Mechanisms of post-transcriptional regulation by microRNAs: are the answers in sight? Nat Rev Genet 2008 9 2 102 114 18197166 10.1038/nrg2290

[B34] Doench JG Sharp PA Specificity of microRNA target selection in translational repression Genes Dev 2004 18 5 504 511 10.1101/gad.1184404 15014042 PMC374233

[B35] Yao J Wang Y Wang W Wang N Li H Solexa sequencing analysis of chicken pre-adipocyte microRNAs Biosci Biotechnol Biochem 2011 75 1 54 61 10.1271/bbb.100530 21228487

[B36] Kwon C Han Z Olson EN Srivastava D MicroRNA1 influences cardiac differentiation in Drosophila and regulates Notch signaling Proc Natl Acad Sci U S A 2005 102 52 18986 18991 10.1073/pnas.0509535102 16357195 PMC1315275

[B37] Clop A Marcq F Takeda H Pirottin D Tordoir X Bibé B Bouix J Caiment F Elsen JM Eychenne F Larzul C Laville E Meish F Milenkovic D Tobin J Charlier C Georges M A mutation creating a potential illegitimate microRNA target site in the myostatin gene affects muscularity in sheep Nat Genet 2006 38 7 813 818 10.1038/ng1810 16751773

[B38] Chen JF Mandel EM Thomson JM Wu Q Callis TE Hammond SM Conlon FL Wang DZ The role of microRNA-1 and microRNA-133 in skeletal muscle proliferation and differentiation Nat Genet 2006 38 2 228 233 10.1038/ng1725 16380711 PMC2538576

[B39] Ichimura A Ruike Y Terasawa K Tsujimoto G miRNAs and regulation of cell signaling FEBS J 2011 278 10 1610 1618 10.1111/j.1742-4658.2011.08087.x 21395975

[B40] Huntzinger E Izaurralde E Gene silencing by microRNAs: contributions of translational repression and mRNA decay Nat Rev Genet 2011 12 2 99 110 10.1038/nrg2936 21245828

[B41] Glazov EA Cottee PA Barris WC Moore RJ Dalrymple BP Tizard ML A microRNA catalog of the developing chicken embryo identified by a deep sequencing approach Genome Res 2008 18 6 957 964 10.1101/gr.074740.107 18469162 PMC2413163

[B42] Kozomara A Griffiths-Jones S miRBase: integrating microRNA annotation and deep-sequencing data Nucleic Acids Res 2011 39 Database issue D152 D157 21037258 10.1093/nar/gkq1027 PMC3013655

[B43] Roush S Slack FJ The let-7 family of microRNAs Trends Cell Biol 2008 18 10 505 516 10.1016/j.tcb.2008.07.007 18774294

[B44] Reinhart BJ Slack FJ Basson M Pasquinelli AE Bettinger JC Rougvie AE Horvitz HR Ruvkun G The 21-nucleotide let-7 RNA regulates developmental timing in Caenorhabditis elegans Nature 2000 403 6772 901 906 10.1038/35002607 10706289

[B45] Ruby JG Jan C Player C Axtell MJ Lee W Nusbaum C Ge H Bartel DP Large-scale sequencing reveals 21U-RNAs and additional microRNAs and endogenous siRNAs in C. elegans Cell 2006 127 6 1193 1207 10.1016/j.cell.2006.10.040 17174894

[B46] Boyerinas B Park SM Hau A Murmann AE Peter ME The role of let-7 in cell differentiation and cancer Endocr Relat Cancer 2010 17 1 F19 F36 10.1677/ERC-09-0184 19779035

[B47] Yang SJ Xu CQ Wu JW Yang GS SOCS3 inhibits insulin signaling in porcine primary adipocytes Mol Cell Biochem 2010 345 1–2 45 52 20683642 10.1007/s11010-010-0558-7

[B48] Sabio G Das M Mora A Zhang Z Jun JY Ko HJ Barrett T Kim JK Davis RJ A stress signaling pathway in adipose tissue regulates hepatic insulin resistance Science 2008 322 5907 1539 1543 10.1126/science.1160794 19056984 PMC2643026

[B49] Bolstad BM Irizarry RA Astrand M Speed TP A comparison of normalization methods for high density oligonucleotide array data based on variance and bias Bioinformatics 2003 19 2 185 193 10.1093/bioinformatics/19.2.185 12538238

[B50] Kanehisa M The KEGG database Novartis Found Symp 2002 247 91 101 12539951

[B51] Kanehisa M Goto S Hattori M Aoki-Kinoshita KF Itoh M Kawashima S Katayama T Araki M Hirakawa M From genomics to chemical genomics: new developments in KEGG Nucleic Acids Res 2006 34 Database issue D354 D357 16381885 10.1093/nar/gkj102 PMC1347464

