# Supplementary material for: Let-7b regulates the expression of the growth hormone receptor gene in deletion-type dwarf chickens
Source: BMC Genomics. 2012 Jul 10;13:306. doi: 10.1186/1471-2164-13-306 (PMC3428657; doi:10.1186/1471-2164-13-306)
Supplement: Additional file 1 — Table S1. The skeletal muscle mRNA differential profile of 14-day-old embryos and 7-week-old chickens of normal chickens and dwarf chickens. [file 1471-2164-13-306-S1.doc]

**Additional Data**

Table S1. The skeletal muscle mRNA differential profiles of 14-day-old embryos and 7-week-old chickens as compared normal with dwarf chickens

| Development stage | **Gene Symbol** | |
| --- | --- | --- |
|  | Up-regulated （**Fold Change**≥2.00） | Down-regulated （**Fold Change≤0**. 50） |
| 14-day-old embryo | *CAMP*, *LECT2*, *GAL1*, *GAL7*, *GAL2*, *TYRP1*, ***LOC770114***, *LYG2*, *NPY*, *GAL6*, *LOC418543*, *LOC425001*, *LOC431317*, *ATG12*, *COL4A2*, *LOC415795*, ***BEAN***, ***HSCB***, *C14orf153*, *AKR7A2*, *LOC425324*, *LOC422305*, ***ARNT***, *RSFR*, *LOC425324 , LOC426333*, *LOC396194 , RSFR*, *LOC771168*, *C1orf58*, *RANGAP1*, *TMEM220*, *C1QB*, ***RCJMB04_1j22****, ATP2A1* | *CDCA8*, *NDUFB4*, *SERTAD2*, *LOC426058*, *FBXL10*, *AHNAK2*, *RCJMB04_10c4*, *NOLA1*, *MOBKL1A*, *LIMS1, LOC771176*, ***LOC772190***, *SLC31A1*, *ANKRD15*, *SLC35B3*, *RHAG*, *TCF25*, *LYAR*, ***TMEM70***, ***GHR***, *RCJMB04_2c16*, *LOC769366*, *WDR12* |
| 7-week-old chickens | *SUCLG2,* ***LOC770114****, RCJMB04_1f9, ACY1L2, LOC776458, OTOR, ENPP4, CA5B, RCJMB04_35g11, VNN1, PON2, FGF1, LOC417943, KLHL38, KCTD20, ASB2, SLC27A1, LOC419322, IGF2BP3, ASB4, P2RX5, KLHL30, CPZ, LOC396260, CHMP6, LOC769739, AIFM2, PTGES, WHDC1, FOXK2, FYCO1, POSTN, PERP, TNFSF10, GFPT2, NINJ1, LOC423162, ALDH1L2, GATSL1, ITGBL1, FBXO22, RCJMB04_35f9, LOC769676 , PLAGL1, FAM69B, FBXO30, TGIF1, LGALS3,* ***BEAN****, CHRND, LAP3, ZFAND2A, RBM7, FAAH, ANKRD9, HLA-G , LOC417056, LOC417083 , MR1 , YFVI, FHL3, RORA,* ***ARNT****, MPP1, EPB41L3, HPGD, CBFB, MAPRE2,* ***HSCB****,* ***RCJMB04_1j22****, EXOSC10, PDGFD, SESN1, HOXA3, USP24, COG1, COMP, RCJMB04_7i20, KLHDC8B, FZD2, RILP, NT5DC1, C4orf16, ARRDC1, CUTA, EEPD1, KLHDC1, LYPLA3, DOK5, PPL, GDPD5, SQSTM1, LOC422757, ABCA3, MYBPH, TRIM63, LOC417013, RCJMB04_16e8, C16orf70, LOC425916, LOC430580, ASH2L, RNF113A, LOC417722, CYP3A80, NUFIP1, TWISTNB, RCJMB04_13a9, PSME4, SCARA5, LOC424014, MAFF, TWIST2, AGTR1* | *CRELD2, HLF, C16orf68, LOC770634, NT5C1A, LOC423138, LMCD1, C11orf52, AGXT2L1, MAGI1, LRP3, DNAJA4, FKBP5, F3, HSP90AA1,* ***LOC772190****, ZNF366, C14orf153, YPEL2, SNAPC5, KCNJ15, DOCK9, THRSP, RGS5, ACOT9, JMJD6, ICER, ST6GAL1, GJA1, MYLK4, DUSP13, BEST3, NR1D2, SLC25A29, PTN, SRD5A2L2, LOC770869, SOCS3, RAB3IP, POP5, LOC777379,* ***TMEM70****, RHOG, LOC423536, KIT, HNMT, VIP, SAMHD1, MB, RCJMB04_1d1, MAT1A, RCJMB04_15h2, HSPH1, CHAC1, HSPA5, BCL6, BATF3, MYH6, HSPA8, CRISPLD2, AANAT, OSGIN1, NPTX2,* ***GHR****, SLC25A30* |

Note: Fold Change ≥ 2.00 means that the expression is up-regulated as compared normal with dwarf chickens. Fold Change ≤ 0.50 indicates the expression was down-regulated as compared normal with dwarf chickens.
