# Supplementary material for: Let-7b regulates the expression of the growth hormone receptor gene in deletion-type dwarf chickens
Source: BMC Genomics. 2012 Jul 10;13:306. doi: 10.1186/1471-2164-13-306 (PMC3428657; doi:10.1186/1471-2164-13-306)
Supplement: Additional file 2 — Table S2. The summary table of prediction results of the differentially expressed. [file 1471-2164-13-306-S2.doc]

Table S2. The summary table of prediction results of the differentially expressed miRNA target genes

| miRNA ID | Target Scan prediction results of target genes |
| --- | --- |
| let-7b | 256 pieces of conservative target gene, including 297 conservative target sites and 20 non-conservative target sites |
| miR-15c, miR-16, miR-16c | 415 pieces of conservative target gene, including 454 conservative target sites and 97 non-conservative target sites |
| miR-17-5p, miR-20a, miR-20b, miR-106 | 479 pieces of conservative target gene, including517 conservative target sites and 72 non-conservative target sites |
| miR-21 | 86 pieces of conservative target gene, including 90 conserved target sites and 13 non- conservative target sites |
| miR-24 | 99 pieces of conservative target gene, including 100 conservative target sites and 10 non-conservative target sites |
| miR-30a-5p, miR-30d, miR-30b, miR-30c | 547 pieces of conservative target gene, including 604 conservative target sites and 124 non-conserved target site |
| miR-92 | 455 pieces of conservative target gene, including 505 conservative target sites and 72 non-conservative target sites |
| miR-99a, miR-100 | 23 pieces of conservative target gene, including 23 conservative target sites |
| miR-126 | 5 pieces of conservative target gene, including five conserved target sites |
| miR-130b | 437 pieces of conservative target gene, including 474 conservative target sites and 81 non-conservative target sites |
| miR-133a, miR-133b, miR-133c | 132 pieces of conservative target gene, including 134 conservative target sites and 10 non-conservative target sites |
| miR-146b | 34 pieces of conservative target gene, including 35 conservative target sites and 5 non-conservative target sites |
| miR-181b | 480 pieces of conservative target gene, including conservative 550 target sites and 116 non-conserved target sites |
| miR-199* | 154 pieces of conservative target gene, including 164 conservative target sites and 23 non-conservative target sites |
| miR-203 | 212 pieces of conservative target gene, including 229 conservative target sites and 80 non-conservative target sites |
| miR-205a | 132 pieces of conservative target gene, including 135 conservative target sites and 31 non-conservative target sites |
| miR-206 | 265 pieces of conservative target gene, including 282 conservative target sites and 34 non-conservative target sites |
| miR-451 | 6 pieces of conservative target gene, including 6 conservative target sites |
