# Supplementary material for: Let-7b regulates the expression of the growth hormone receptor gene in deletion-type dwarf chickens
Source: BMC Genomics. 2012 Jul 10;13:306. doi: 10.1186/1471-2164-13-306 (PMC3428657; doi:10.1186/1471-2164-13-306)
Supplement: Additional file 3 — Table S3. The intersection genes of the differentially expressed genes of miRNA target genes and mRNA genes. [file 1471-2164-13-306-S3.doc]

Table S3. The intersection genes of the differentially expressed genes of miRNA target genes and mRNA genes

| miRNA ID | Intersection genes |
| --- | --- |
| let-7b | *GHR, IGF2BP3, YPEL2* |
| miR-15c*,* miR-16*,* miR-16c | *FKBP5, GHR, HOXA3, MYLK4* |
| miR-17-5p*,* miR-20a*,* miR-20b*,* miR-106 | *C16orf70, FBXO30, HLF, HSPA8* |
| miR-21 | *C4orf16* |
| miR-24 | *MAGI1, SESN1* |
| miR-30a-5p*,* miR-30d*,* miR-30b*,* miR-30c | *BCL6, GJA1, HLF, HSPA5, SOCS3, USP24, YPEL2* |
| miR-92 | *GFPT2, TGIF1* |
| miR-130b | *C16orf70, CBFB, HLF, HOXA3, HSPA8, KIT* |
| miR-133a*,* miR-133b*,* miR-133c | *YPEL2* |
| miR-181b | *C4orf16, EPB41L3, FOXK2, GHR, HLF, HSPA5, IGF2BP3, USP24* |
| miR-199* | *HLF, HSPA5* |
| miR-203 | *FOXK2, KCNJ15* |
| miR-205a | *MAGI1, VIP* |
| miR-206 | *HOXA3* |
