# Supplementary material for: Let-7b regulates the expression of the growth hormone receptor gene in deletion-type dwarf chickens
Source: BMC Genomics. 2012 Jul 10;13:306. doi: 10.1186/1471-2164-13-306 (PMC3428657; doi:10.1186/1471-2164-13-306)
Supplement: Additional file 4 — Figure S1. The JAK-STAT signaling pathway with GHR gene involved in KEGG links the genome information with gene function. The pathway includes 111 genes in total. [file 1471-2164-13-306-S4.doc]

Table S4. Sequences of primers used for qRT-PCR

| Genes | Sequence | Annealing temperature  (°C) | Product  Amplicon size (bp) | Accession No. |
| --- | --- | --- | --- | --- |
| GHR | F：5'GCAAGTGCAGGTCACCTGAG3'  R：5'CCGGACATTCTTTCCAGTCT3' | 58.8 | 126 | NM_001001293.1 |
| SOCS3 | F：5'CCAGTCTGGGTTACTGCACATCA3'  R：5'ATTCCACCATGGCATCACGA3' | 60 | 106 | NM_204600.1 |
| IRS1 | F：5'CTTCGGTGTCTGGTTCCC3'  R：5'ATAGTTGCTTAGCTCCTCCTCA3' | 60 | 155 | NM_001031570.1 |
| PI3K | F：5'GGAATGAATGGCTGTCGTATGAC3'  R：5'CCAATGGACAGTGCTCCTCTTTA3' | 60 | 120 | NM_001004410.1 |
| LEPR | F：5'TACCGTGCCTACTGCTGA3'  R：5'AACTGGCGTTGTTATTGC3' | 56 | 168 | NM_204323.1 |
| PPARA | F：5'ACGGAGTTCCAATCGC3'  R：5'AACCCTTACAACCTTCACAA3' | 57 | 220 | NM_001001464.1 |
| JAK2 | F：5'CAAACCTCCTTGTCTTCA3'  R：5'CTCCCACTTCTTTCCTTAT3' | 51.9 | 183 | NM_001030538.1 |
| STAT3 | F：5'GGAGGCATTTGGGAAGTA3'  R：5'ATGGGCAGGTCAATGGTA3' | 56.3 | 141 | NM_001030931.1 |
| MYOD1 | F：5'GGCCGCCGATGACTTCTATG 3'  R：5'GCGTTGGTGGTCTTCCTCTTG 3' | 62.7 | 243 | NM_204214.1 |
| MyoG | F：5'GGCTTTGGAGGAGAAGGACT3'  R：5'CAGAGTGCTGCGTTTCAGAG3' | 56.9 | 184 | NM_204184.1 |
| Myf5 | F：5'GGAGGAGGCTGAAGAAAGTGA3'  R：5'TCTGTCCCGGCAGGTGAT3' | 61.7 | 177 | NM_001030363.1 |
| IGF2BP3 | F：5'GCTGCTGCTGCTTCATATCCAC3'  R：5'CCTGCTTGCCAATAATAGCTCCA3' | 60 | 103 | NM_001006359.1 |
| IGF-1 | F：5'TGGCCTGTGTTTGCTTACCTT3'  R：5'TACGAACTGAAGAGCATCAACCA3' | 60 | 91 | NM_001004384.2 |
| β-actin | F：5'CCCCATGCCATCCTCCGTCTG3'  R：5'CCTCGGGGCACCTGAACCTCTC3' | 61 | 265 | NM_205518 |
