# Supplementary material for: Let-7b regulates the expression of the growth hormone receptor gene in deletion-type dwarf chickens
Source: BMC Genomics. 2012 Jul 10;13:306. doi: 10.1186/1471-2164-13-306 (PMC3428657; doi:10.1186/1471-2164-13-306)
Supplement: Additional file 5 — Figure S2. Adipocytokine signaling pathway with the SOCS3 gene involved in. [file 1471-2164-13-306-S5.doc]

Table S5. Sequences of primers used for vectors construction

| Genes | Primer Sequence | Annealing temperature  (°C) | Product  Amplicon Length  (bp) |
| --- | --- | --- | --- |
| Pre-let-7b | F:5'ATT***GCGGCCGC***TGCCTGTAGAGTCACCCCACC3'  R:5'GGT***GGGCCC***CCAGAAACAAAACAAATCAAGAAC3' | 61 | 541 |
| GHR-3’UTR | F:5'ATG***GAGCTC***CTCTTTGCCTAACAGCATCTCCT3'  R:5'CGT***TCTAGA***GCAATAAACACATTCTGTCGGG3' | 60 | 752 |
