# Supplementary material for: Let-7b regulates the expression of the growth hormone receptor gene in deletion-type dwarf chickens
Source: BMC Genomics. 2012 Jul 10;13:306. doi: 10.1186/1471-2164-13-306 (PMC3428657; doi:10.1186/1471-2164-13-306)

Figure S1. The JAK-STAT signaling pathway with *GHR* gene involved in KEGG links the genome information with gene function. The pathway includes 111 genes in total.


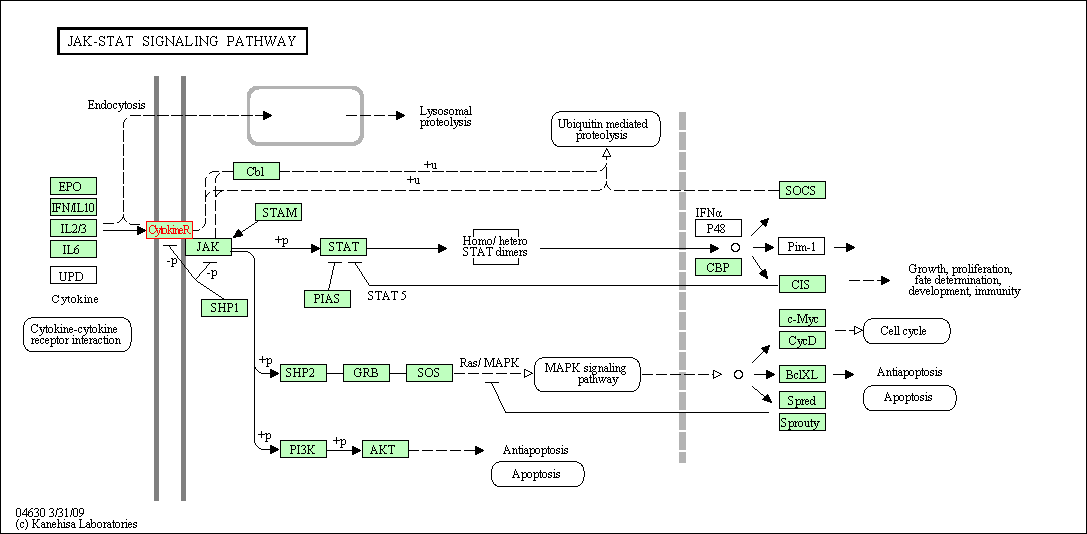

Supplement: Additional file 6 — Table S4. Sequences of primers used for qRT-PCR. [file 1471-2164-13-306-S6.doc]
