# Supplementary figures and images for: Let-7b regulates the expression of the growth hormone receptor gene in deletion-type dwarf chickens
Source: BMC Genomics. 2012 Jul 10;13:306. doi: 10.1186/1471-2164-13-306 (PMC3428657; doi:10.1186/1471-2164-13-306)

Figure S2. Adipocytokine signaling pathway with the *SOCS3* gene involved in.


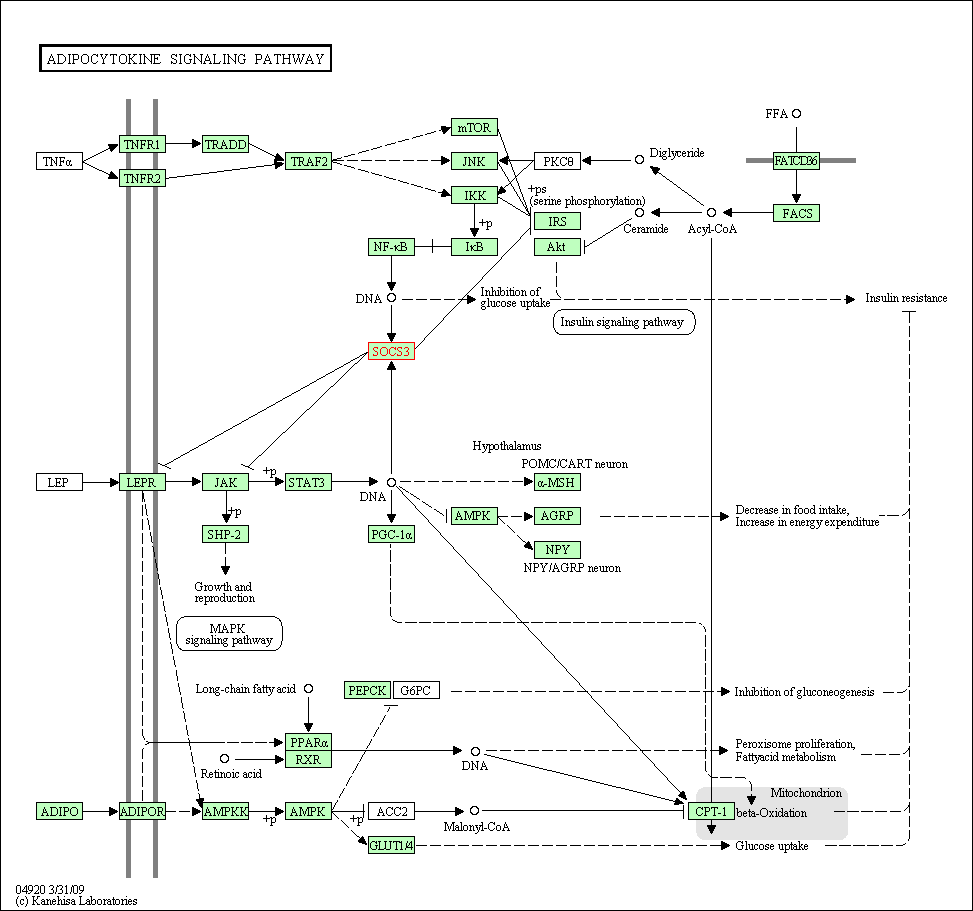

Supplement: Additional file 7 — Table S5. Sequences of primers used for vectors construction. [file 1471-2164-13-306-S7.doc]
